# Wogonin Suppresses Non-Small Cell Lung Cancer Growth in Association with Oxidative Stress, c-Myc/GPX4 Downregulation and Ferroptosis-Related Responses

**DOI:** 10.3390/antiox15070891

**Published:** 2026-07-19

**Authors:** Hairong Xiang, Haoshu Liu, Ruyu Jiang, Xiaomeng Tang, Linfeng Zhao, Dawei Zeng, Yue Zhang, Jiazhen Xie, Liangqin Shi, Lan Yang

**Affiliations:** 1 College of Basic Medicine, Chengdu University of Traditional Chinese Medicine, Chengdu 611137, China; xianghairong1@stu.cdutcm.edu.cn (H.X.); liuhaoshu@cdzyydx1.wecom.work (H.L.); jiangruyu@stu.cdutcm.edu.cn (R.J.); zhaolinfeng1@stu.cdutcm.edu.cn (L.Z.); zengdawei911@outlook.com (D.Z.); zhangyue2001yy@outlook.com (Y.Z.); jiazhen18796@outlook.com (J.X.); 2 School of Pharmacy, Chengdu University of Traditional Chinese Medicine, Chengdu 611137, China; tangxiaomeng@stu.cdutcm.edu.cn

**Keywords:** Wogonin, reactive oxygen species, oxidative stress, antioxidant defense, non-small cell lung cancer, ferroptosis, mitochondrial apoptosis, c-Myc/GPX4

## Abstract

Reactive oxygen species (ROS)-regulated antioxidant defense is closely linked to non-small cell lung cancer (NSCLC) progression and therapy resistance. Wogonin (WGN), a flavonoid from *Scutellaria baicalensis*, has antitumor activity, but whether it is associated with ROS-dependent ferroptotic and mitochondrial stress in NSCLC remains incompletely defined. A549 and BEAS-2B cells, male BALB/c nude mouse A549 xenografts, patient-derived NSCLC organoids, and public transcriptomic cohorts were analyzed using viability, colony formation, migration/invasion, DCFH-DA ROS, JC-1, Annexin V/PI, Fe^2+^ and lipid ROS probes, RT-qPCR, Western blotting, immunofluorescence, inhibitor rescue, and c-Myc gain- and loss-of-function assays. WGN suppressed A549 growth and motility with weaker effects on BEAS-2B cells. WGN markedly increased intracellular ROS, Fe^2+^ accumulation and lipid peroxidation, decreased mitochondrial membrane potential, promoted Caspase-related apoptosis, reduced c-Myc/GPX4 and SLC7A11, and increased ACSL4. N-acetylcysteine, Z-VAD-FMK and Ferrostatin-1 partially rescued WGN-induced injury. c-Myc overexpression partially restored GPX4 and reduced lipid ROS/Fe^2+^ accumulation, whereas c-Myc knockdown decreased GPX4. Xenografts and organoids reproduced tumor inhibition and selected redox-associated molecular changes. Collectively, WGN suppresses A549-associated NSCLC phenotypes in association with ROS accumulation, ferroptosis-related lipid injury, mitochondrial dysfunction-associated apoptosis, and c-Myc/GPX4 downregulation.

## 1. Introduction

Lung cancer is one of the malignancies with the highest incidence and mortality worldwide, and non-small cell lung cancer (NSCLC) accounts for the major proportion of lung cancer cases [1,2]. In recent years, targeted therapy and immune checkpoint inhibitors have improved outcomes in selected patients, but primary non-response, acquired resistance, recurrence, metastasis, and treatment-related toxicity continue to restrict long-term benefit [3,4,5]. A central feature of NSCLC stress adaptation is the ability to buffer reactive oxygen species (ROS) and preserve redox homeostasis under oncogenic, metabolic, and therapeutic pressure. Tumor cells can therefore survive by remodeling antioxidant defense, mitochondrial function, anti-apoptotic signaling, and microenvironmental adaptation. Strategies that convert this adaptive redox buffering into lethal oxidative injury may provide a useful approach for overcoming stress tolerance in NSCLC.

Ferroptosis is a regulated form of cell death distinct from apoptosis, necrosis, and autophagy, and is characterized by iron-dependent lipid peroxide accumulation, ROS amplification, and membrane oxidative damage [6,7]. During this process, expansion of the intracellular labile iron pool allows Fe^2+^ to promote ROS generation through the Fenton reaction, while phospholipids containing polyunsaturated fatty acids undergo peroxidation and ultimately compromise membrane integrity. From a redox-biology perspective, ferroptosis reflects a failure of antioxidant lipid-peroxide defense rather than a simple increase in total ROS. Tumor cells often depend strongly on iron metabolism, lipid synthesis, and antioxidant systems, making them potentially vulnerable to interventions that disrupt ROS buffering and lipid peroxide clearance [8,9,10,11,12,13,14].

The SLC7A11/GPX4 axis is an important antioxidant defense system against ferroptosis [15,16]. SLC7A11, as a key component of the cystine/glutamate antiporter system, promotes cystine uptake and helps maintain glutathione synthesis. GPX4 uses reduced glutathione to eliminate phospholipid hydroperoxides, thereby limiting the propagation of lipid peroxidation and protecting membranes from ROS-driven oxidative collapse. ACSL4 promotes the activation of polyunsaturated fatty acids and participates in membrane phospholipid remodeling, providing a substrate basis for lipid peroxidation; it is therefore commonly regarded as a facilitator of ferroptosis sensitivity [17,18].

c-Myc is a key oncogenic transcription factor involved in tumor cell proliferation, metabolic reprogramming, mitochondrial function, and oxidative stress adaptation [19,20,21,22]. In ferroptosis research, the role of c-Myc is not unidirectional. It may increase oxidative pressure by enhancing metabolic activity, while it may also support tumor cell adaptation by maintaining antioxidant or proliferation-related programs. Thus, in specific drug and tumor contexts, the relationship between c-Myc alterations and downstream ferroptosis-associated molecules needs to be defined through functional experiments.

*Scutellaria baicalensis* Georgi (Lamiaceae; Huang-Qin) is a representative traditional medicinal herb frequently used in heat-clearing and detoxification prescriptions. Its major flavonoids include baicalin, baicalein, and WGN [23]. WGN is a natural flavonoid derived from *S. baicalensis* and exhibits anti-inflammatory, immunomodulatory, redox-regulatory, and antitumor activities [23]. As a redox-active flavonoid, WGN is particularly relevant to antioxidant and oxidative-stress research because its biological effect may depend on cellular context: in normal cells it may support redox balance, whereas in tumor cells with high basal oxidative demand it may amplify ROS stress and weaken antioxidant adaptation. Previous work also reported that WGN promoted ferroptosis-related changes in pancreatic cancer cells through Fe^2+^ accumulation, lipid peroxidation, GSH depletion, and Nrf2/GPX4 modulation [24]. Because inflammation, oxidative stress, and redox adaptation are closely connected with NSCLC progression and treatment resistance, evaluating WGN-induced ROS accumulation, lipid peroxidation, ferroptosis-related responses, and mitochondrial apoptosis provides a mechanistically focused strategy for interpreting its modern antitumor value.

Against this background, the present study used A549 cells, BEAS-2B normal bronchial epithelial cells, A549 nude mouse xenografts, and patient-derived NSCLC organoids as the principal models, together with public transcriptomic analyses. Following a ROS-centered sequence of phenotypic inhibition, oxidative stress measurement, mitochondrial injury, lipid-peroxide accumulation, ferroptosis-related antioxidant defense, pharmacological rescue, c-Myc functional validation, and in vivo and organoid support, this study evaluated whether WGN is associated with a shift in A549 cells from redox adaptation toward ROS-associated ferroptosis-related and apoptotic injury.

## 2. Materials and Methods

### 2.1. Reagents and Antibodies

Wogonin (WGN; P105403), a purified flavonoid constituent associated with *Scutellaria baicalensis Georgi*, was purchased from Chengdu Lingliu Biotechnology Co., Ltd. (Chengdu, China). Its molecular formula is C_16_H_12_O_5_, with a molecular weight of 284.27 and a purity of ≥98%. Because the present study used a commercially purified compound rather than collected plant material or an in-house herbal extract, no voucher specimen was generated. WGN was dissolved in DMSO to prepare a stock solution, aliquoted, and stored at −80 °C. In the basic in vitro treatment experiments, the working concentrations of WGN were set at 0, 5, 10, 20, and 40 μM, and the final concentration of DMSO did not exceed 0.1%. The Vehicle group received an equal volume of DMSO. Fer-1 (MedChemExpress, Monmouth Junction, NJ, USA; HY-100579; 10 μM, 2 h pretreatment), Z-VAD-FMK (Beyotime Biotechnology, Shanghai, China; C1202; 20 μM, 1 h pretreatment), and NAC (MedChemExpress, Monmouth Junction, NJ, USA; HY-B0215; 5 mM, 2 h pretreatment) were used for rescue experiments related to ferroptosis, Caspase-related apoptosis, and ROS-related injury, respectively. The Annexin V-FITC/PI apoptosis kit was purchased from Biosharp (Beijing, China; BL107B), DCFH-DA from Solarbio (Beijing, China; CA1410), RhoNox-6 from Beyotime Biotechnology (Shanghai, China; S1070S), JC-1 (Solarbio, Beijing, China; M8650), and BODIPY 581/591 C11 from Beyotime Biotechnology (Shanghai, China; S0043S). The primary antibodies included GPX4 (Proteintech Group, Rosemont, IL, USA; 67763-1-Ig, 1:2000), SLC7A11 (Solarbio, Beijing, China; K115345P, 1:2000), ACSL4 (ImmunoWay Biotechnology, Plano, TX, USA; YM8287, 1:2000), c-Myc (ImmunoWay Biotechnology, Plano, TX, USA; YM8143, 1:2000), E-cadherin (1:2000), Cleaved Caspase-3 (1:1000), Cleaved Caspase-9 (1:1000), GAPDH (1:5000), β-actin (1:5000), Ki67 (1:200), and PCNA (1:200).

### 2.2. Cell Culture and Drug Treatment

The human lung cancer cell line A549 and the normal human bronchial epithelial cell line BEAS-2B were obtained from the American Type Culture Collection (ATCC, Manassas, VA, USA; A549, CCL-185; BEAS-2B, CRL-3588). A549 cells were cultured in RPMI-1640 medium (VivaCell, Shanghai, China), and BEAS-2B cells were cultured in DMEM (VivaCell, Shanghai, China). Both media were supplemented with 10% fetal bovine serum (ExCell Bio, Suzhou, China), 100 μg/mL streptomycin and 100 U/mL penicillin (Solarbio, Beijing, China). Cells were maintained in an incubator at 37 °C with 5% CO_2_ and saturated humidity. Logarithmically growing cells were seeded into 96-well plates, 6-well plates, culture dishes, Transwell chambers, or fluorescence imaging plates according to the specific assay. After cell attachment, different concentrations of WGN were administered. In rescue experiments, cells were pretreated with NAC, Z-VAD-FMK, or Fer-1 before the addition of WGN for further incubation. A549 was selected as a well-characterized lung adenocarcinoma model suitable for redox, ferroptosis-related, and xenograft analyses; the limitations associated with its specific redox background are addressed in Section 4.

### 2.3. Cell Viability Assay

Cell viability was measured using the CCK-8 assay. A549 or BEAS-2B cells were seeded into 96-well plates at 8 × 10^3^ cells/well. After overnight attachment, cells were treated with 0, 5, 10, 20, or 40 μM WGN for 24 h or 48 h. After treatment, CCK-8 working solution was added and incubated in the dark, and absorbance was read at 450 nm. Relative cell viability was calculated as the ratio of the absorbance in the treatment group to that in the Vehicle group.

### 2.4. Rescue Viability Assay

In rescue viability assays, A549 cells were pretreated with Fer-1 (10 μM) for 2 h, Z-VAD-FMK (20 μM) for 1 h, or NAC (5 mM) for 2 h, followed by treatment with gradient concentrations of WGN. At the end of treatment, absorbance at 450 nm was measured according to the CCK-8 kit instructions to evaluate the contributions of ferroptosis, Caspase-related apoptosis, and ROS to WGN-induced cytotoxicity.

### 2.5. Colony Formation Assay

A549 cells were seeded into 6-well plates at 500 cells/well. After overnight attachment, the cells were treated with different concentrations of WGN for 48 h. The medium was then replaced every 3 days, and the cells were cultured for another 10–14 days. When visible colonies had formed (≥50 cells per colony), culture was terminated. Colonies were fixed with 4% paraformaldehyde and stained with 0.5% crystal violet. After air drying, images were acquired and colonies were automatically counted using Fiji/ImageJ (version 1.54u7; National Institutes of Health, Bethesda, MD, USA).

### 2.6. Wound-Healing Assay

A549 cells were seeded into 6-well plates at 5 × 10^5^ cells/well and cultured to 90–100% confluence. A sterile 10 μL pipette tip was used to generate a straight scratch. Floating cells were removed by washing, after which low-serum medium containing the corresponding concentrations of WGN was added. Cells were fixed and imaged at 0, 24, and 48 h. Before imaging, cells were fixed with 4% paraformaldehyde and stained with 0.5% crystal violet. Scratch width was measured using Fiji/ImageJ (version 1.54u7; National Institutes of Health, Bethesda, MD, USA), and the relative migration distance was calculated.

### 2.7. Transwell Migration and Invasion Assays

For the migration assay, 200 μL of A549 cell suspension (1 × 10^4^ cells) was added to the upper chamber of an 8 μm-pore Transwell insert (Corning, Corning, NY, USA; 353097). For the invasion assay, Matrigel (Corning, Corning, NY, USA; 356234) was diluted 1:8 with serum-free medium, and 50 μL was added to the upper chamber and allowed to solidify before 200 μL of A549 cell suspension (2 × 10^4^ cells) was added. The lower chamber contained 600 μL of medium supplemented with 20% fetal bovine serum. After treatment with gradient concentrations of WGN for 48 h, cells were fixed with 4% paraformaldehyde for 30 min, stained with 0.1% crystal violet for 15 min, and counted in six randomly selected fields under a light microscope.

### 2.8. Annexin V/PI Apoptosis Assay

After treatment with different concentrations of WGN for 48 h, A549 cells were collected together with the culture supernatant. The adherent cells were digested with EDTA-free trypsin and combined with the supernatant. After centrifugation at 2000 rpm for 5 min, the cells were washed twice with PBS and resuspended in 500 μL of Binding Buffer. Subsequently, 5 μL of Annexin V-FITC and 5 μL of PI were added, and the cells were incubated at room temperature in the dark for 5–15 min. Detection was performed within 1 h using a BD FACSCanto II flow cytometer (BD Biosciences, Franklin Lakes, NJ, USA).

### 2.9. ROS Detection

Intracellular ROS levels were detected using the DCFH-DA fluorescent probe. After A549 cells were treated with different concentrations of WGN for 48 h, the culture medium was removed and DCFH-DA working solution was added. Cells were incubated at 37 °C in the dark for 30 min. After washing three times with serum-free medium, cells were collected and fluorescence signals were detected using the PE channel of a BD FACSCanto II flow cytometer (BD Biosciences, Franklin Lakes, NJ, USA; Ex/Em = 561/585 nm). ROS fluorescence changes were also observed using an ImageXpress Micro Confocal high-content imaging system (Molecular Devices, San Jose, CA, USA).

### 2.10. Mitochondrial Membrane Potential Assay

Mitochondrial membrane potential was assessed by JC-1 staining. After A549 cells were treated with different concentrations of WGN for 48 h, JC-1 staining working solution was added and cells were incubated at 37 °C for 20 min. The working solution was removed, cells were washed twice with JC-1 staining buffer, and an appropriate amount of serum-free medium was added. Red and green fluorescence signals were observed using an ImageXpress Micro Confocal high-content imaging system (Molecular Devices, San Jose, CA, USA), and fluorescence quantification was performed with Fiji/ImageJ (version 1.54u7; National Institutes of Health, Bethesda, MD, USA). A decrease in the red/green fluorescence ratio indicated loss of mitochondrial membrane potential.

### 2.11. Detection of Fe^2+^ Accumulation and Lipid Peroxidation

Intracellular Fe^2+^ levels were detected using the RhoNox-6 probe. RhoNox-family probes are reaction-based Fe^2+^-selective fluorescent probes in which Fe^2+^-mediated deoxygenation of the N-oxide group increases fluorescence, thereby allowing detection of the labile ferrous iron pool [25]. After A549 cells were treated with different concentrations of WGN for 48 h, they were digested with EDTA-free trypsin, collected, and incubated with RhoNox-6 working solution at 37 °C in the dark for 30 min. Fluorescence intensity was then measured using the PE channel of a BD FACSCanto II flow cytometer (BD Biosciences, Franklin Lakes, NJ, USA; Ex/Em = 561/585 nm). Lipid peroxidation was detected using the BODIPY 581/591 C11 probe. BODIPY 581/591 C11 is a membrane-incorporated oxidation-sensitive lipid peroxidation probe whose fluorescence shifts from red to green after oxidation, enabling assessment of lipid ROS/lipid peroxide accumulation [26]. After treatment and collection, cells were incubated with the staining working solution at 37 °C in the dark for 30 min, followed by quantitative analysis using a BD FACSCanto II flow cytometer (BD Biosciences, Franklin Lakes, NJ, USA).

### 2.12. Western Blotting

Total cellular protein was extracted using RIPA lysis buffer (Biosharp, Beijing, China; BL504A), and protein concentrations were determined using a BCA protein assay kit (Beyotime Biotechnology, Shanghai, China; P0010). Protein samples were separated by SDS-PAGE and transferred onto PVDF membranes (Merck, Darmstadt, Germany; ISEQ00010). After blocking with 5% skim milk, membranes were incubated with primary antibodies overnight at 4 °C. Primary antibodies were used at the following dilutions unless otherwise stated: c-Myc, GPX4, SLC7A11, ACSL4, and E-cadherin at 1:2000; Cleaved Caspase-3 and Cleaved Caspase-9 at 1:1000; GAPDH and β-actin at 1:5000. Membranes were then incubated with horseradish peroxidase-conjugated secondary antibodies at 1:5000 for 1 h at room temperature. Signals were acquired using a Tanon 6100 chemiluminescence imaging system (Tanon Science & Technology, Shanghai, China), and band intensities were quantified with Fiji/ImageJ (version 1.54u7; National Institutes of Health, Bethesda, MD, USA).

### 2.13. RT-qPCR

Total RNA was extracted from A549 cells using TRIZOL® Reagent (Thermo Fisher Scientific, Waltham, MA, USA; 1559026CN), and RNA was reverse-transcribed into cDNA using HiScript III RT SuperMix (Vazyme Biotech, Nanjing, China; R323-01). The reverse transcription program was as follows: 42 °C for 2 min, 37 °C for 15 min, and 85 °C for 5 s. Real-time quantitative PCR was then performed according to the instructions of ChamQ Universal SYBR qPCR Master Mix (Vazyme Biotech, Nanjing, China; Q711-02/03). The amplification program was as follows: pre-denaturation at 95 °C for 10 min; 40 cycles of 95 °C for 15 s and 60 °C for 45 s; and a melting curve program of 95 °C for 15 s, 60 °C for 1 min, 95 °C for 15 s, and 60 °C for 15 s. GAPDH mRNA served as the internal control, and relative target gene expression was calculated using the 2^−ΔΔCt^ method. RT-qPCR primer sequences are listed in Supplementary Materials Table S1.

c-Myc overexpression and knockdown experiments were performed according to the instructions for the EZ Trans transfection reagent (Shanghai Life-iLab Biotech Co., Ltd., Shanghai, China; AC04L010). The c-Myc overexpression plasmid was provided by GenePharma Biotechnology (Shanghai, China), and the overexpression vector was pcDNA3.1-C. The culture medium was replaced 6 h after transfection, and cellular status was observed using a fluorescence microscope (Nikon, Tokyo, Japan; TI-FL). Cells were then cultured for another 24 h and collected for subsequent experiments. The efficiencies of c-Myc overexpression and knockdown were both verified by qRT-PCR. The c-Myc siRNA oligonucleotide sequences are listed in Supplementary Materials Table S2. Based on preliminary comparison, MYC-Homo-621 showed higher c-Myc knockdown efficiency and was therefore selected for subsequent c-Myc knockdown experiments.

### 2.14. Immunofluorescence

Cells or tissue sections were fixed with 4% paraformaldehyde, permeabilized with 0.2% Triton X-100, blocked with blocking solution, and incubated with primary antibodies overnight at 4 °C. For cellular immunofluorescence, antibodies against c-Myc, GPX4, and ACSL4 were used at 1:200. For tissue immunofluorescence, PCNA antibody was used at 1:200. For immunohistochemistry, antibodies against Ki67, E-cadherin, c-Myc, and GPX4 were used at 1:200. Samples were then incubated with fluorescence-labeled secondary antibodies at 1:500 or corresponding IHC secondary reagents at room temperature for 1 h, and nuclei were counterstained with DAPI where applicable. After mounting with anti-fade reagent, images were acquired using an ImageXpress Micro Confocal high-content imaging system (Molecular Devices, San Jose, CA, USA), and Fiji/ImageJ (version 1.54u7; National Institutes of Health, Bethesda, MD, USA) was used for image processing and fluorescence quantification.

### 2.15. Bioinformatics Analyses

Public transcriptomic analyses included NSCLC cohorts such as GSE19188, GSE19804, GSE30219, and GSE31210. Differential expression between tumor and normal tissues was assessed using the Wilcoxon test. Spearman correlation analysis was performed with MYC as the anchor to evaluate correlations between MYC and GPX4 as well as genes associated with ferroptosis, oxidative stress/lipid peroxidation, and mitochondrial apoptosis. Tumor samples in the GSE30219 cohort were divided into GPX4-high and GPX4-low groups according to the median GPX4 expression level, and Ferroptosis_Driver, ROS_Response, Lipid_Peroxidation, and Migration_Invasion functional scores were compared. The Kaplan–Meier method was used for overall survival analysis, and Cox proportional hazards models were applied to evaluate the associations of MYC, GPX4, and Ferroptosis_Tendency_Net with clinical outcomes.

### 2.16. Xenograft Model and Histological Analysis

All animal experiments were performed according to a protocol approved by the Animal Ethics Committee of Chengdu University of Traditional Chinese Medicine (approval no. 2025806) and were reported in accordance with ARRIVE principles. Procedures complied with institutional guidelines and the National Research Council Guide for the Care and Use of Laboratory Animals. Male BALB/c nude mice aged 6–8 weeks were purchased from Chengdu Yaokang Biotechnology Co., Ltd. (Chengdu, China); because only male mice were used, sex-based differences were not evaluated in this xenograft model. Mice were randomly assigned to the Vehicle and WGN groups, with six mice in each group. Each mouse was subcutaneously injected in the right forelimb region with 100 μL of cell suspension containing 5 × 10^6^ A549 cells. After tumors reached the preset volume, the WGN group received intraperitoneal WGN at 60 mg/kg/d for 8 consecutive days. Body weight was recorded daily during treatment. Tumor length, defined as the longest diameter, and tumor width, defined as the longest perpendicular diameter, were measured with digital calipers during treatment, and tumor volume was calculated using the formula: tumor volume = length × width^2^/2. At the experimental endpoint, mice were euthanized, and tumor tissues were excised, weighed, photographed, fixed, and embedded. Tissue sections were used for H&E staining, immunohistochemistry, and immunofluorescence. Ki67, E-cadherin, c-Myc, and GPX4 were detected by IHC, while PCNA was detected by tissue immunofluorescence.

### 2.17. Patient-Derived Organoid Culture and Drug Sensitivity Assay

Patient-derived NSCLC organoids were used under institutional ethical requirements for human-derived materials. Written informed consent was obtained for the use of de-identified tumor-derived samples, and donor privacy rights were observed through de-identification before organoid culture and analysis. Organoids were embedded in MasterAim matrix gel (AimingMed, Hangzhou, China) and cultured in MasterAim complete organoid medium (AimingMed, Hangzhou, China) for three-dimensional culture. Organoids were seeded into 384-well plates at a density of 1500 cells per well. When organoid density exceeded 80%, organoids were treated with different concentrations of WGN for 72 h. Organoid viability was measured using an ATP-based chemiluminescence assay (MasterAim Organoid Viability ATP Assay Kit, AimingMed, Hangzhou, China). Fixed organoids were subjected to immunofluorescence staining for c-Myc, GPX4, and ACSL4 to evaluate the effects of WGN in a three-dimensional clinically relevant model.

### 2.18. Statistical Analysis

In vitro cell-based experiments, organoid experiments, and molecular quantifications were analyzed using independent biological replicates as the statistical unit; technical replicates were not counted as independent biological replicates. Quantitative analyses were derived from at least three independent experiments. In animal experiments, each mouse was used as the statistical unit, and the A549 nude mouse xenograft experiment included six mice in each group. Unless otherwise stated in the figure legends, data are presented as mean ± standard deviation; continuously monitored data such as tumor volume curves are presented as mean ± standard error according to the figure legends. Statistical analyses and graphing were performed using GraphPad Prism 10.0. Two-group comparisons were performed using a two-tailed Student’s *t*-test, and multiple-group comparisons were performed using one-way ANOVA followed by Tukey’s post hoc test. Tumor volume and body-weight curves were analyzed using repeated-measures analysis. Public transcriptomic correlations were evaluated using Spearman’s method, survival curves were analyzed by the log-rank test, and Cox proportional hazards models were used to evaluate survival-related variables. *p* < 0.05 was considered statistically significant, and significance is indicated in the corresponding figures.

## 3. Results

### 3.1. Wogonin Suppresses NSCLC Cell Growth and Motility with Weaker Effects on BEAS-2B Cells

**Figure 1:**
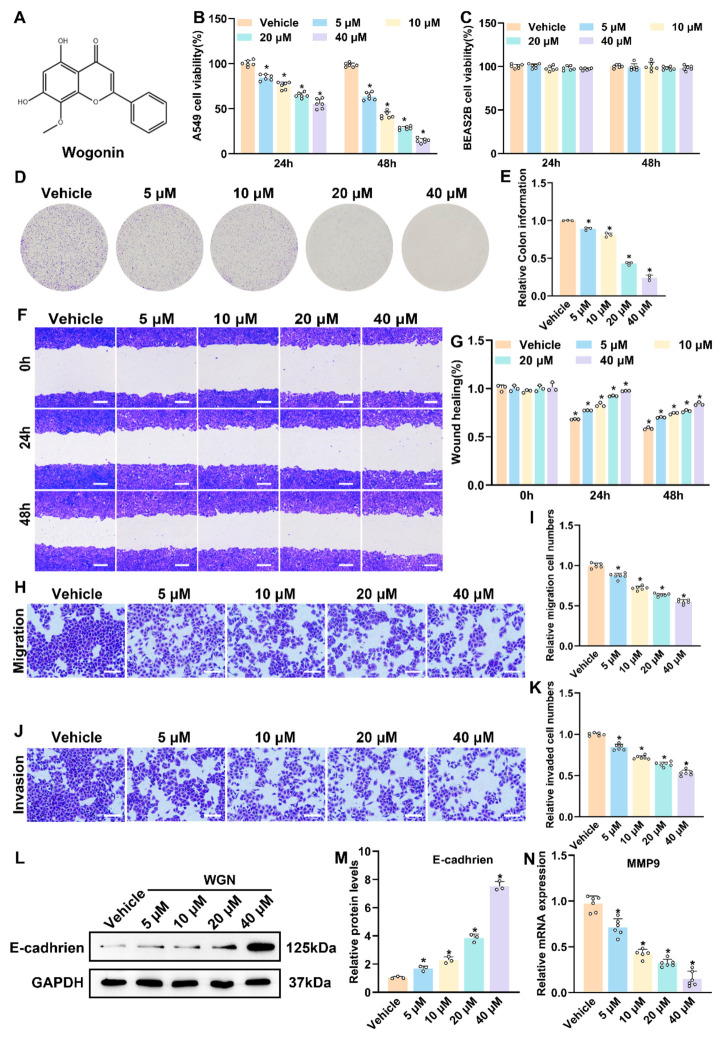
Wogonin suppresses A549 cell proliferation, migration, and invasion and exerts a weaker effect on BEAS-2B cells under the same treatment conditions. (**A**): Chemical structure of Wogonin. (**B**): Viability of A549 cells after treatment with 0, 5, 10, 20, and 40 μM WGN for 24 h and 48 h. (**C**): Viability of BEAS-2B cells after treatment with the same concentrations of WGN. (**D**,**E**): Colony-forming capacity of A549 cells after treatment with different concentrations of WGN. (**F**,**G**): Wound-healing changes in A549 cells treated with different concentrations of WGN for 0, 24, and 48 h. Scale bar: 100 μm. (**H**,**I**): Transwell migration of A549 cells after treatment with different concentrations of WGN for 48 h. Scale bar: 100 μm. (**J**,**K**): Matrigel-coated Transwell invasion of A549 cells after treatment with different concentrations of WGN for 48 h. Scale bar: 100 μm. (**L**,**M**): E-cadherin protein expression detected by Western blotting. (**N**): MMP9 mRNA expression detected by RT-qPCR. Data are presented as mean ± SD; quantitative analyses were derived from at least three independent experiments; one-way ANOVA followed by Tukey’s post hoc test; * *p* < 0.05 compared with the Vehicle group.

To determine the direct inhibitory effect of WGN on NSCLC cells (chemical structure shown in Figure 1A), CCK-8 assays were first performed to measure A549 cell viability after treatment with different concentrations of WGN for different durations. The results showed that WGN reduced A549 cell viability in a concentration-dependent manner after both 24 h and 48 h of treatment, with a more pronounced inhibitory effect after 48 h (Figure 1B). Under the same concentration conditions, BEAS-2B cell viability decreased to a lesser extent (Figure 1C), suggesting that WGN exerted a more prominent inhibitory effect on tumor cells under the tested conditions. Colony formation assays further showed that WGN treatment reduced the number of A549 cell colonies, indicating suppression of long-term proliferative capacity (Figure 1D,E). Wound-healing assays showed that A549 cells in the Vehicle group exhibited obvious wound closure during the observation period, whereas WGN treatment slowed wound closure (Figure 1F,G). Transwell assays likewise showed that WGN treatment decreased the numbers of migrated and invaded cells (Figure 1H–K). Molecular assays showed that WGN upregulated E-cadherin protein expression and decreased MMP9 mRNA expression (Figure 1L–N), suggesting that the inhibitory effect on migration and invasion may be associated with enhanced cell adhesion and reduced extracellular matrix degradation.

### 3.2. Wogonin Promotes ROS Accumulation and ROS-Associated Mitochondrial Dysfunction and Apoptosis

**Figure 2:**
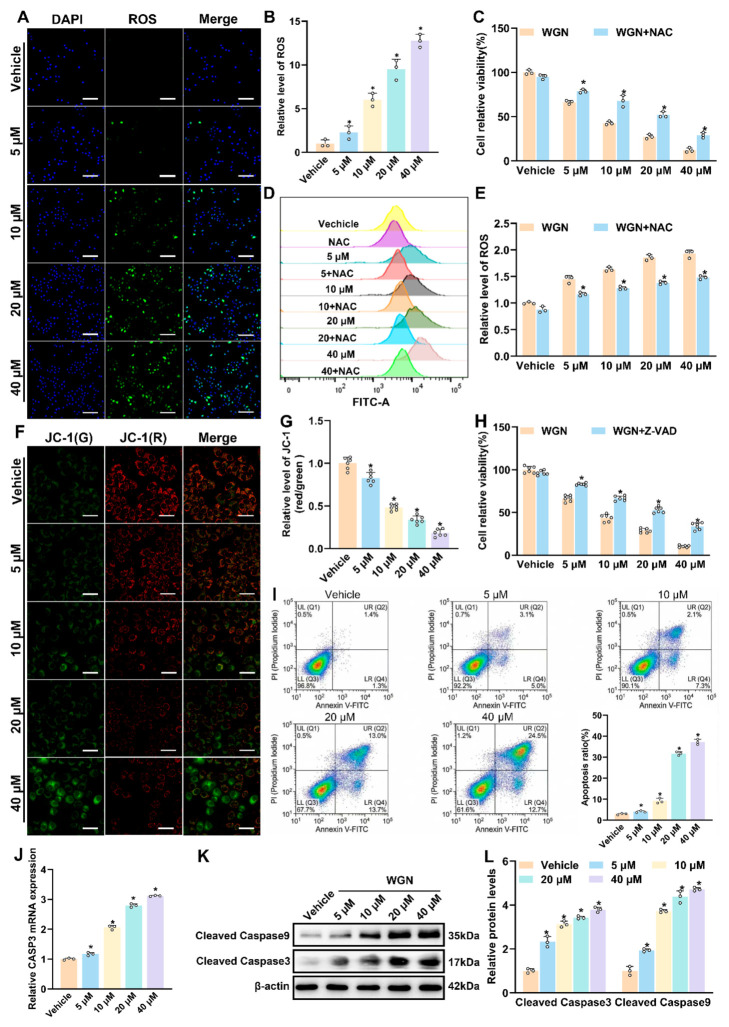
Wogonin induces ROS accumulation, mitochondrial membrane potential loss, and Caspase-dependent apoptosis in A549 cells. (**A**,**B**): Intracellular ROS levels detected by DCFH-DA staining after A549 cells were treated with 0, 5, 10, 20, and 40 μM WGN for 48 h. Scale bar: 100 μm. (**C**): Effects of NAC pretreatment (5 mM, 2 h) combined with different concentrations of WGN on cell viability. (**D**,**E**): Effects of NAC pretreatment (5 mM, 2 h) on WGN-induced ROS signal changes detected by flow cytometry. (**F**,**G**): Mitochondrial membrane potential detected by JC-1 staining after A549 cells were treated with different concentrations of WGN for 48 h, followed by JC-1 staining for 20 min. Scale bar: 100 μm. In the fluorescence images, blue indicates DAPI-stained nuclei, green indicates DCF fluorescence or JC-1 monomers, and red indicates JC-1 aggregates; merged images show channel colocalization. (H): Effects of Z-VAD-FMK pretreatment (20 μM, 1 h) combined with different concentrations of WGN on cell viability. (**I**): Cell apoptosis after WGN treatment detected by Annexin V-FITC/PI flow cytometry. (**J**): CASP3 mRNA expression detected by RT-qPCR. (**K**,**L**): Cleaved Caspase-3 and Cleaved Caspase-9 protein expression detected by Western blotting. Data are presented as mean ± SD; quantitative analyses were derived from at least three independent experiments; one-way ANOVA followed by Tukey’s post hoc test; * *p* < 0.05 compared with the Vehicle group or the indicated group.

After confirming that WGN suppressed the malignant phenotype of A549 cells, we next examined whether this response was accompanied by disruption of intracellular redox homeostasis. DCFH-DA fluorescence imaging and flow cytometry showed that WGN markedly increased intracellular ROS levels in a concentration-dependent manner (Figure 2A,B,D,E). NAC pretreatment reduced WGN-induced ROS signals and partially restored cell viability (Figure 2C), indicating that ROS accumulation is not merely a bystander signal but a functional contributor to WGN-induced injury. JC-1 staining showed decreased red fluorescence and increased green fluorescence after WGN treatment, with a reduced red/green fluorescence ratio, indicating loss of mitochondrial membrane potential (Figure 2F,G). MitoTracker detection in the Supplementary Materials further showed that WGN treatment decreased mitochondrial membrane potential (Figure S1A,B), consistent with the JC-1 results and jointly supporting ROS-associated mitochondrial dysfunction. Annexin V/PI flow cytometry showed an increased proportion of apoptotic cells after WGN treatment (Figure 2I), while Z-VAD-FMK partially alleviated the WGN-induced decrease in cell viability (Figure 2H). Increased CASP3 mRNA and Cleaved Caspase-3 and Cleaved Caspase-9 protein levels (Figure 2J–L) further supported Caspase-related apoptosis associated with ROS-linked mitochondrial dysfunction.

### 3.3. Wogonin Disrupts Antioxidant Lipid-Peroxide Defense and Elicits Ferroptosis-Related Alterations

**Figure 3:**
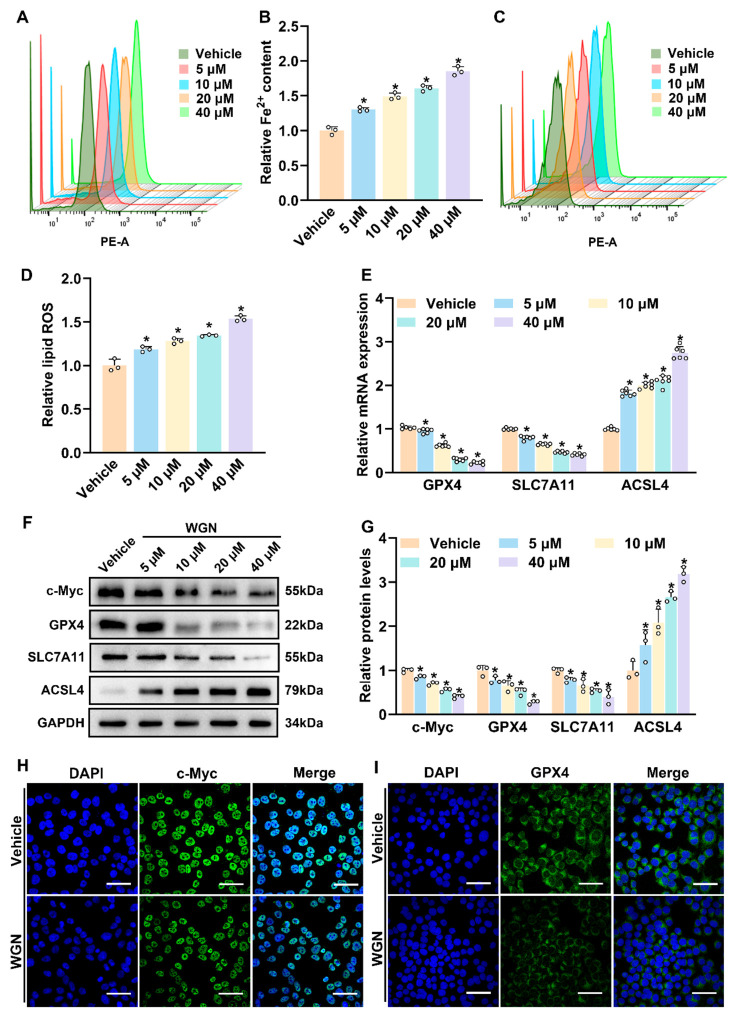
Wogonin disrupts ferroptosis-related antioxidant defense in A549 cells. (**A**,**B**): Intracellular Fe^2+^ levels detected by RhoNox-6 flow cytometry after A549 cells were treated with 0, 5, 10, 20, and 40 μM WGN for 48 h. (**C**,**D**): Lipid peroxidation/lipid ROS levels detected by C11-BODIPY 581/591 flow cytometry after A549 cells were treated with different concentrations of WGN for 48 h. (**E**): GPX4, SLC7A11, and ACSL4 mRNA expression detected by RT-qPCR. (**F**,**G**): c-Myc, GPX4, SLC7A11, and ACSL4 protein expression detected by Western blotting. (**H**,**I**): c-Myc and GPX4 protein signals detected by immunofluorescence. Scale bar: 100 μm. Data are presented as mean ± SD; quantitative analyses were derived from at least three independent experiments; one-way ANOVA followed by Tukey’s post hoc test; * *p* < 0.05 compared with the Vehicle group.

The core features of ferroptosis include Fe^2+^ accumulation, enhanced lipid peroxidation, and collapse of antioxidant lipid-peroxide defense. RhoNox-6 flow cytometry showed that WGN treatment increased intracellular Fe^2+^ levels in A549 cells (Figure 3A,B). BODIPY 581/591 C11 detection showed enhanced lipid ROS/lipid peroxidation signals after WGN treatment (Figure 3C,D). Together, Fe^2+^ accumulation and lipid peroxidation indicated that WGN shifted A549 cells toward a ROS-amplifying ferroptosis-related state. Further analysis of ferroptosis-related molecules showed that WGN reduced the mRNA expression of GPX4 and SLC7A11 while increasing ACSL4 mRNA expression (Figure 3E). GPX4 is a central lipid peroxide-detoxifying enzyme that suppresses ferroptosis by reducing phospholipid hydroperoxides. SLC7A11 supports cystine uptake and glutathione synthesis, thereby helping maintain GPX4-mediated antioxidant capacity. ACSL4 promotes incorporation of polyunsaturated fatty acids into membrane phospholipids and increases susceptibility to lipid peroxidation. At the protein level, WGN decreased c-Myc, GPX4, and SLC7A11 expression and increased ACSL4 expression (Figure 3F,G). Immunofluorescence showed weakened c-Myc and GPX4 protein signals in WGN-treated A549 cells (Figure 3H,I), consistent with the Western blot results. These results suggest that WGN is associated with impaired c-Myc/GPX4-related antioxidant defense, enhanced lipid ROS accumulation, and Fe^2+^-associated oxidative injury in A549 cells.

### 3.4. Ferrostatin-1 Partially Rescues Wogonin-Induced Ferroptosis-Related Lipid Injury

**Figure 4:**
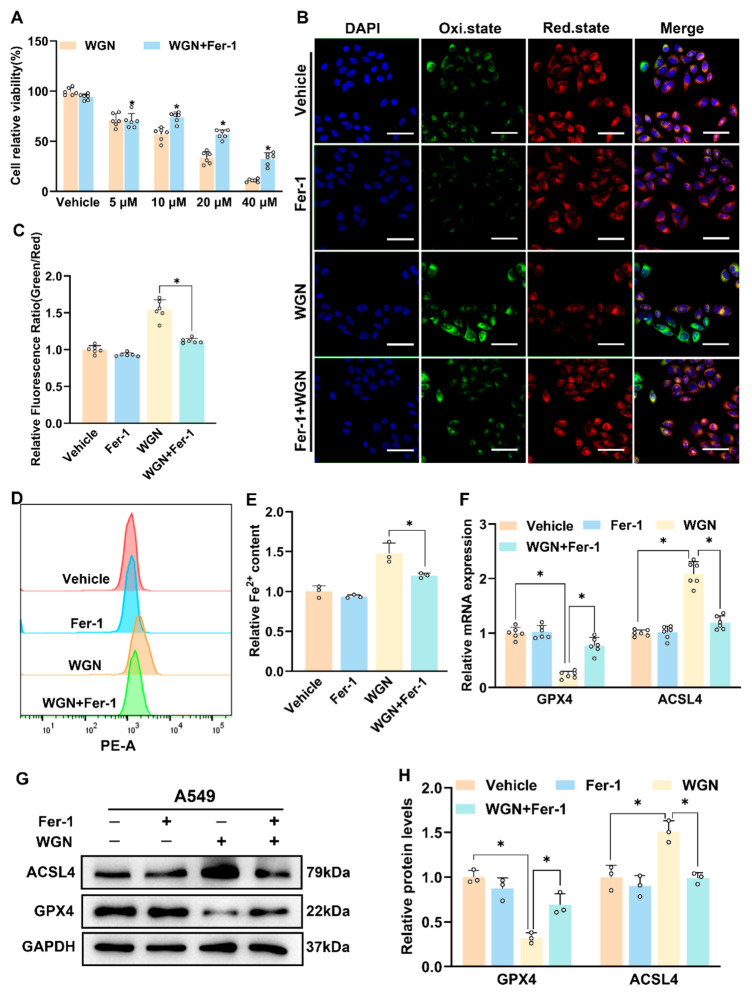
Fer-1 partially reverses WGN-induced ferroptosis-related phenotypes and related molecular changes. (**A**): Effects of Fer-1 pretreatment combined with different concentrations of WGN on A549 cell viability. (**B**,**C**): Effects of Fer-1 on WGN-induced lipid peroxidation detected by C11-BODIPY 581/591. Scale bar: 100 μm. (**D**,**E**): Effects of Fer-1 on WGN-induced Fe^2+^ accumulation detected by RhoNox-6 flow cytometry. (**F**): GPX4 and ACSL4 mRNA expression detected by RT-qPCR. (**G**,**H**): GPX4 and ACSL4 protein expression detected by Western blotting. Data are presented as mean ± SD; quantitative analyses were derived from at least three independent experiments; one-way ANOVA followed by Tukey’s post hoc test; * *p* < 0.05 between the indicated groups.

To further evaluate the functional contribution of ferroptosis-related lipid peroxidation to WGN-induced cytotoxicity, Fer-1 was used for pharmacological rescue. Fer-1 is a radical-trapping antioxidant and ferroptosis inhibitor that suppresses lipid-peroxidation chain reactions and protects membranes from oxidative lipid damage [27]. The results showed that Fer-1 pretreatment partially restored the WGN-induced decrease in cell viability (Figure 4A), indicating that lipid-peroxidation chain reactions participate in WGN-mediated cellular injury. BODIPY 581/591 C11 detection showed that Fer-1 reduced WGN-induced lipid peroxidation signals (Figure 4B,C). RhoNox-6 detection also showed that Fer-1 partially decreased Fe^2+^-related fluorescence signals (Figure 4D,E). Molecular analyses further showed that Fer-1 to some extent reversed the WGN-induced decrease in GPX4 and increase in ACSL4 (Figure 4F–H). The partial, rather than complete, rescue by Fer-1, together with the partial rescue by Z-VAD-FMK, suggests that WGN-induced injury involves ferroptosis-related lipid peroxidation and Caspase-related apoptosis rather than a single death pathway.

### 3.5. c-Myc Gain- and Loss-of-Function Assays Support a GPX4-Associated Redox-Defense Response to Wogonin

**Figure 5:**
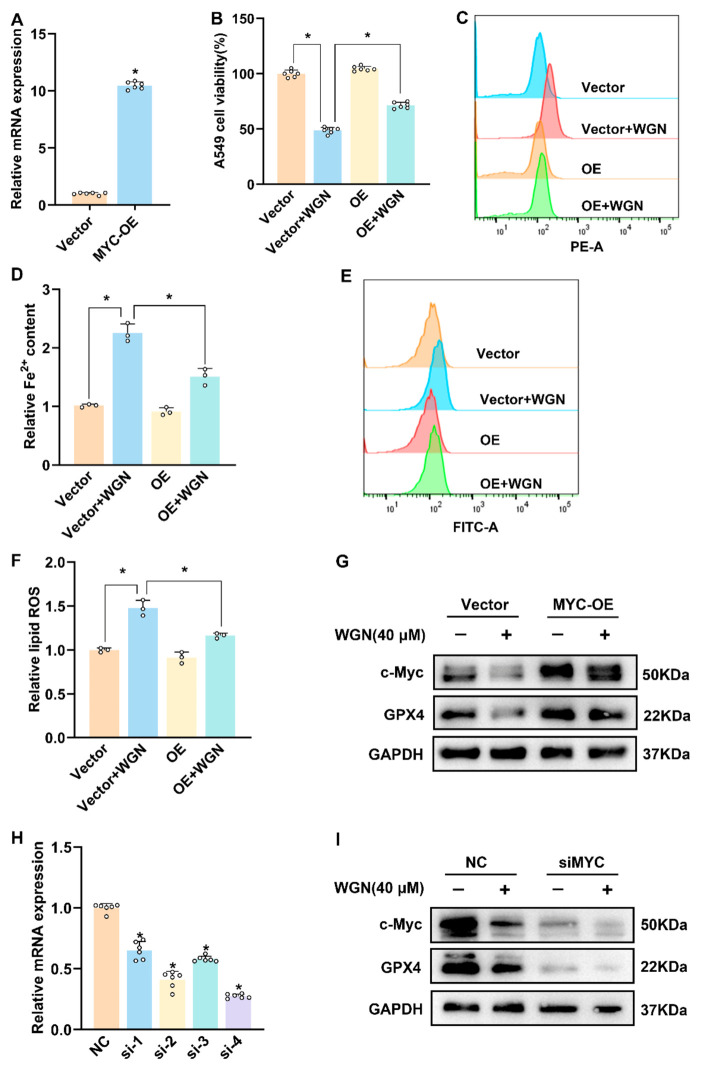
c-Myc overexpression or knockdown modulates WGN-induced ferroptotic phenotypes and GPX4 expression. (**A**): RT-qPCR validation of c-Myc overexpression efficiency. (**B**): Effects of c-Myc overexpression on WGN-induced loss of cell viability detected by CCK-8 assay. (**C**,**D**): Effects of c-Myc overexpression on WGN-induced Fe^2+^ accumulation detected by RhoNox-6 flow cytometry. (**E**,**F**): Effects of c-Myc overexpression on WGN-induced lipid peroxidation detected by C11-BODIPY 581/591 flow cytometry. (**G**): Effects of c-Myc overexpression on WGN-induced changes in GPX4 protein detected by Western blotting. (**H**): RT-qPCR screening of c-Myc siRNA knockdown efficiency. (**I**): c-Myc and GPX4 protein expression after c-Myc knockdown detected by Western blotting. Data are presented as mean ± SD; quantitative analyses were derived from at least three independent experiments; two-tailed Student’s *t*-test was used for two-group comparisons, and one-way ANOVA followed by Tukey’s post hoc test was used for multiple comparisons; * *p* < 0.05 compared with the corresponding control or the indicated group.

Because the decrease in c-Myc protein after WGN treatment occurred together with GPX4 downregulation and enhanced Fe^2+^/lipid ROS phenotypes, c-Myc overexpression and knockdown experiments were further performed to assess its functional relevance in redox defense remodeling. RT-qPCR showed a marked increase in c-Myc mRNA in the overexpression group (Figure 5A). Western blotting showed that, under WGN treatment, c-Myc overexpression partially restored GPX4 protein levels (Figure 5G), suggesting a functional association between decreased c-Myc protein and impaired GPX4-mediated antioxidant protection. Functionally, c-Myc overexpression alleviated the WGN-induced decrease in A549 cell viability and reduced Fe^2+^ accumulation and lipid ROS levels (Figure 5B–F). In contrast, siRNA-mediated c-Myc knockdown reduced c-Myc expression and was accompanied by decreased GPX4 expression (Figure 5H,I), partially mimicking the molecular changes induced by WGN. Taken together, the overexpression and knockdown results indicate that the c-Myc/GPX4-associated axis is functionally related to WGN-induced disruption of antioxidant lipid-peroxide defense in A549 cells, while direct regulation of GPX4 by c-Myc requires further validation.

### 3.6. Public Transcriptomic Analyses Support the Redox and Clinical Relevance of the MYC/GPX4-Associated State in NSCLC

**Figure 6:**
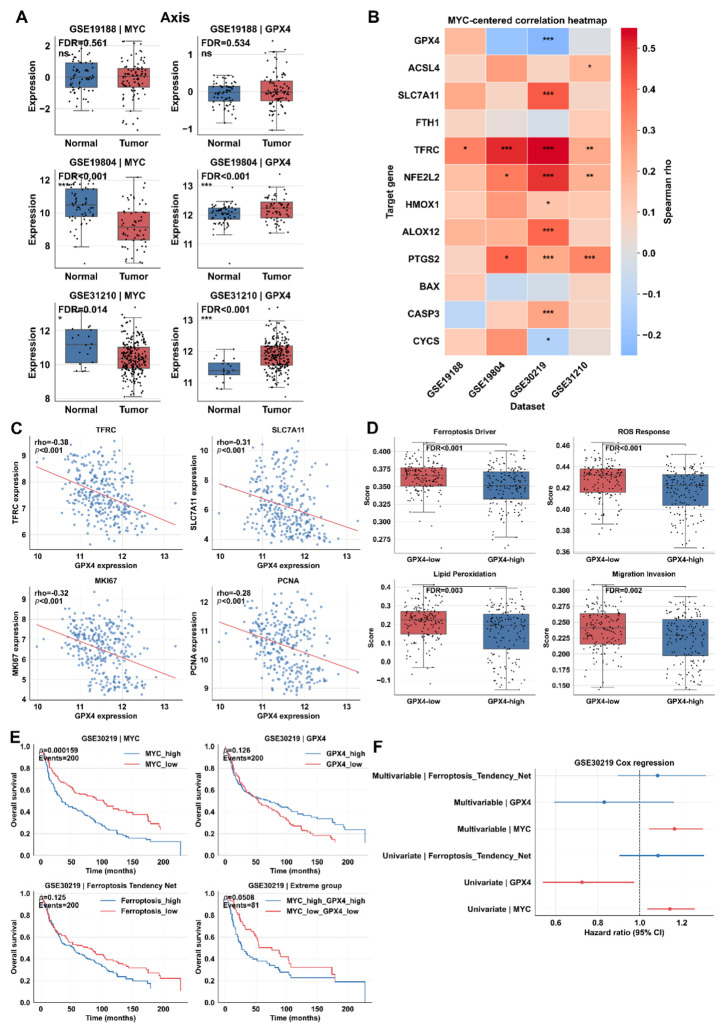
Public transcriptomic analyses support the association of the *MYC*/*GPX4*-related redox-defense state with ferroptotic status and clinical outcomes in NSCLC. (**A**): Tumor-normal expression differences in *MYC* and *GPX4* in the GSE19188, GSE19804, and GSE31210 cohorts. (**B**): Spearman correlation heatmap of key molecules using *MYC* as the anchor. (**C**): Correlations of *GPX4* with *TFRC*, *SLC7A11*, *MKI67*, and *PCNA* in the GSE30219 cohort. (**D**): Differences in Ferroptosis_Driver, ROS_Response, Lipid_Peroxidation, and Migration_Invasion functional scores between *GPX4*-high and *GPX4*-low tumor samples in the GSE30219 cohort. (**E**): Kaplan–Meier overall survival curves stratified by *MYC* expression in the GSE30219 cohort. (**F**): Cox regression forest plot of *MYC*, *GPX4*, and Ferroptosis_Tendency_Net in the GSE30219 cohort. Differential expression was assessed using the Wilcoxon test; correlations were assessed using Spearman’s method; survival differences were assessed using the log-rank test; and the Cox model shows HR and 95% CI. Italic p or FDR values are indicated in the corresponding panels; * *p* < 0.05, ** *p* < 0.01, *** *p* < 0.001, and ns, not significant.

To evaluate the biological significance of the c-Myc/*GPX4*-associated axis in NSCLC from an external redox-biology perspective, public transcriptomic cohorts including GSE19188, GSE19804, GSE30219, and GSE31210 were integrated. Comparisons between tumor and normal tissues showed abnormal expression patterns of *MYC* and *GPX4* in NSCLC tissues (Figure 6A), suggesting a transcriptomic basis for this redox-defense axis. A correlation heatmap anchored on *MYC* showed that *MYC* was associated with *GPX4* and multiple genes related to ferroptosis, oxidative stress/lipid peroxidation, and mitochondrial apoptosis (Figure 6B). In the GSE30219 cohort, *GPX4* correlated with *TFRC*, *SLC7A11*, *MKI67*, and *PCNA* (Figure 6C), suggesting that the *GPX4*-associated state is linked not only to anti-ferroptotic defense but also to tumor proliferative programs. Comparison between *GPX4*-high and *GPX4*-low groups showed differences in Ferroptosis_Driver, ROS_Response, Lipid_Peroxidation, and Migration_Invasion scores (Figure 6D). Kaplan–Meier survival analysis and Cox regression showed that *MYC*, *GPX4*, and Ferroptosis_Tendency_Net were associated to some extent with clinical outcomes in NSCLC (Figure 6E,F). Public transcriptomic results mainly provide supportive evidence for the expression profile, functional scores, and clinical relevance of the *MYC*/*GPX4*-associated redox-defense state; they cannot substitute for direct transcriptomic sequencing after WGN treatment or target-binding experiments.

### 3.7. Wogonin Suppresses Xenograft Growth In Vivo and Reproduces Key Redox-Defense Changes

**Figure 7:**
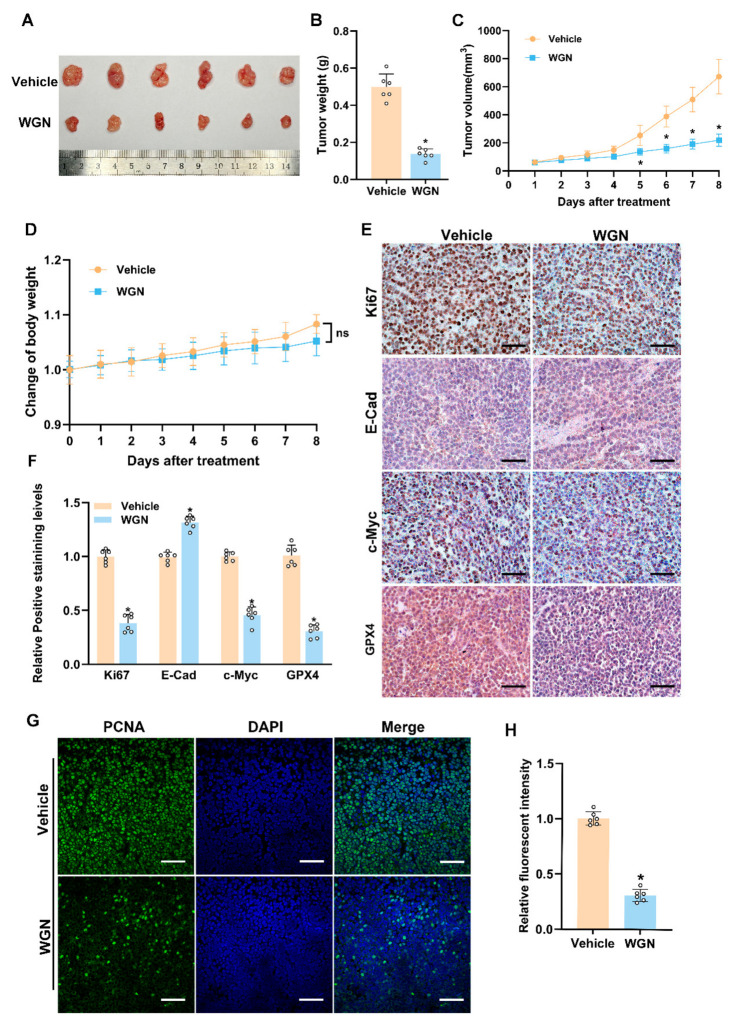
WGN suppresses A549 nude mouse xenograft growth and reproduces key histological changes. (**A**): Images of excised tumors at the experimental endpoint. (**B**): Endpoint tumor weight. (**C**): Tumor volume changes during treatment. (**D**): Mouse body weight changes during treatment. (**E**,**F**): Immunohistochemical detection of Ki67, E-cadherin, c-Myc, and GPX4 in tumor tissues. Scale bar: 100 μm. (**G**,**H**): Immunofluorescence detection of PCNA in tumor tissues. Scale bar: 100 μm. Data are presented as mean ± SD; each group included six mice; two-tailed Student’s *t*-test was used for endpoint measurements, and repeated-measures analysis was used for tumor volume and body weight curves; * *p* < 0.05 compared with the Vehicle group; ns, not significant.

A549 subcutaneous xenografts in nude mice were used to evaluate whether the in vitro redox-associated antitumor response could be reproduced in vivo. Compared with the Vehicle group, the WGN-treated group showed reduced tumor volume and tumor weight at the endpoint (Figure 7A,B), and the tumor growth curve also showed that WGN slowed tumor growth (Figure 7C). No obvious decrease in mouse body weight was observed during treatment, and H&E staining of tumor tissues showed no overt abnormal morphological changes (Figure 7D and Figure S2A), together suggesting that no obvious toxicity signal was observed under the current dosing conditions. Histological and immunostaining results showed reduced Ki67 and PCNA signals in tumor tissues after WGN treatment (Figure 7E–H), indicating that tumor cell proliferation activity was suppressed in vivo. E-cadherin expression was increased, consistent with the decreased migration and invasion observed in vitro (Figure 7E,F). Meanwhile, c-Myc and GPX4 expression levels were reduced in tumor tissues (Figure 7E,F). In the absence of in vivo Fer-1 rescue and quantitative tissue-level lipid-peroxidation measurements, these findings support in vivo antitumor activity accompanied by reduced c-Myc and GPX4 expression, but do not directly establish ferroptosis as the in vivo mechanism.

### 3.8. Wogonin Remains Active in NSCLC Organoids and Recapitulates the Redox-Associated Response Pattern

**Figure 8:**
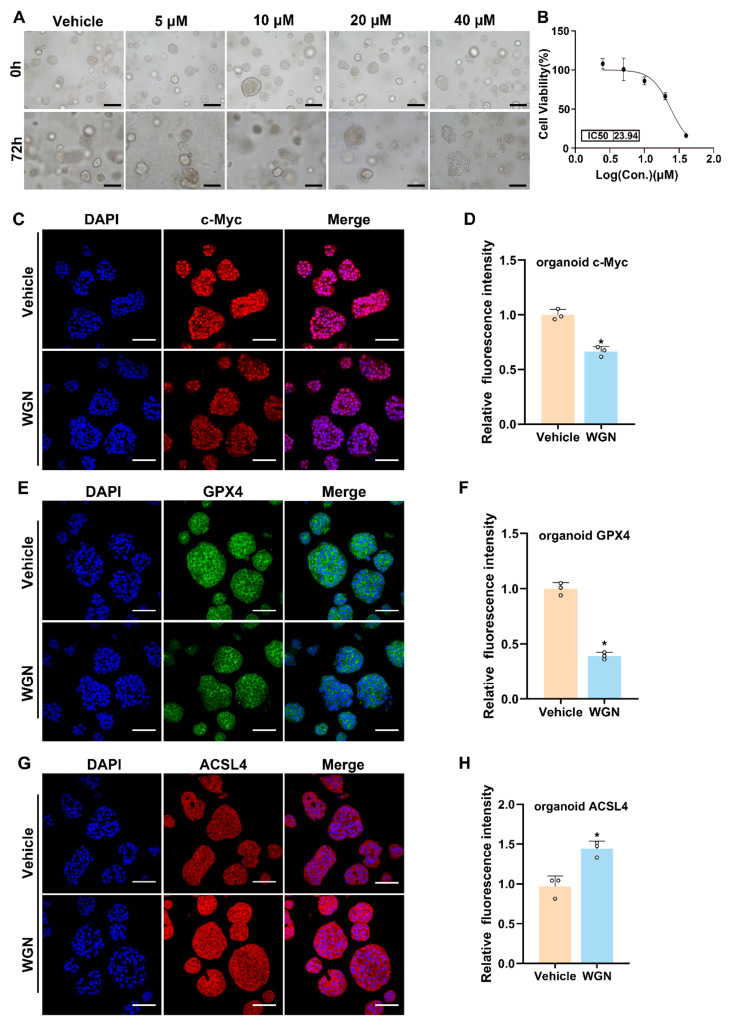
Patient-derived NSCLC organoids validate the antitumor activity and key molecular changes induced by WGN. (**A**): Morphological changes in organoids treated with Vehicle or different concentrations of WGN for 0 h and 72 h. Scale bar: 100 μm. (**B**): Organoid viability after treatment with different concentrations of WGN for 72 h measured by ATP-based bioluminescence. (**C**,**D**): c-Myc expression changes in organoids after WGN treatment detected by immunofluorescence. Scale bar: 100 μm. (**E**,**F**): GPX4 expression changes in organoids after WGN treatment detected by immunofluorescence. Scale bar: 100 μm. (**G**,**H**): ACSL4 expression changes in organoids after WGN treatment detected by immunofluorescence. Scale bar: 100 μm. Data are presented as mean ± SD; quantitative analyses were derived from at least three independent experiments; two-tailed Student’s *t*-test was used for two-group comparisons, and one-way ANOVA followed by Tukey’s post hoc test was used for dose–response comparisons; * *p* < 0.05 compared with the Vehicle group.

Patient-derived NSCLC organoids can to some extent retain tumor spatial architecture and heterogeneity, making them an important complement linking two-dimensional cell experiments with clinically relevant models. Bright-field images showed morphological changes after WGN treatment, including reduced organoid size, loosened structure, and increased fragmentation (Figure 8A). ATP-based bioluminescence assays showed that organoid viability decreased as WGN concentration increased (Figure 8B), indicating that WGN also inhibited tumor cell survival in the three-dimensional organoid model. Immunofluorescence showed that WGN decreased c-Myc and GPX4 expression while increasing ACSL4 expression in organoids (Figure 8C–H). These findings were consistent with the decreased c-Myc/GPX4 and increased ACSL4 observed in the two-dimensional A549 culture system. Because ferroptosis-specific functional rescue assays were not performed in organoids, these data should be interpreted as validation of antitumor activity and selected molecular trends in a three-dimensional model rather than definitive proof of organoid ferroptosis.

### 3.9. Proposed ROS-Centered Working Model of the Wogonin Response in NSCLC

**Figure 9:**
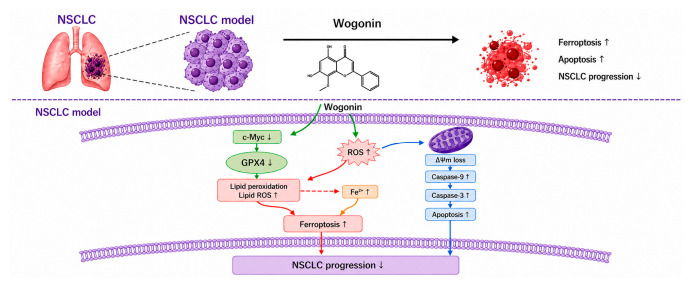
Proposed ROS-centered working model linking WGN treatment with c-Myc/GPX4 downregulation, ferroptosis-related lipid stress, and mitochondrial dysfunction-associated apoptosis in A549 cells. Red arrows indicate the ferroptosis-related lipid-peroxidation branch, blue arrows indicate the mitochondrial apoptosis branch, and green arrows indicate the c-Myc/GPX4 regulatory branch; ↑ denotes an increase or activation, whereas ↓ denotes a decrease or inhibition.

The above findings support a ROS-centered working model (Figure 9): WGN does not induce cellular injury through a single pathway alone, but is associated with ferroptosis-related lipid injury and mitochondrial dysfunction-associated apoptosis under enhanced oxidative stress. In the ferroptosis-related branch, WGN treatment is accompanied by decreased c-Myc and GPX4 expression, increased lipid ROS accumulation, and Fe^2+^-associated oxidative injury. In the apoptosis-related branch, ROS accumulation is accompanied by mitochondrial membrane potential loss, Caspase-related activation, and increased apoptosis. The fact that NAC, Z-VAD-FMK, and Fer-1 each produced only partial rescue suggests that WGN-induced cellular injury may reflect the joint involvement of ROS accumulation, mitochondrial dysfunction-associated apoptosis, and ferroptosis-related lipid oxidative injury.

## 4. Discussion

To our knowledge, this study shows that WGN, a major flavonoid derived from *S. baicalensis*, suppresses A549-associated NSCLC phenotypes in association with ROS accumulation, ferroptosis-related lipid stress, and mitochondrial dysfunction-associated apoptosis. This finding strengthens the connection between the redox-regulatory pharmacology of *S. baicalensis*-derived flavonoids and the modern antitumor mechanism of its purified constituent WGN. Rather than merely showing that WGN inhibits proliferation and motility, the present work links its antitumor activity to intracellular ROS accumulation, c-Myc/GPX4 reduction, lipid ROS elevation, and Fe^2+^-associated ferroptosis-related injury. The main mechanistic implication is that WGN is associated with weakened c-Myc/GPX4-related antioxidant lipid-peroxide defense, while SLC7A11 downregulation and ACSL4 upregulation provide additional support for a ferroptosis-permissive redox state.

Ferroptosis is driven by the convergence of iron availability, lipid peroxide accumulation, ROS amplification, and insufficient antioxidant capacity [6,7,10]. In the present study, WGN increased intracellular ROS, Fe^2+^, and lipid ROS while reducing c-Myc/GPX4-associated protective signaling; SLC7A11 downregulation and ACSL4 upregulation further indicated a ferroptosis-permissive state. These changes are mechanistically coherent: GPX4 limits lipid peroxide propagation through enzymatic detoxification of phospholipid hydroperoxides, whereas the SLC7A11/GSH system supports GPX4 activity and ACSL4 facilitates the formation of oxidizable membrane lipid substrates [15,16,17,18]. Thus, under the present experimental conditions, WGN appears to be associated with weakening of c-Myc/GPX4-associated antioxidant lipid-peroxide defense and with cooperation between ROS/lipid ROS accumulation and Fe^2+^-related oxidative injury. The partial reversal produced by Fer-1 further supports the contribution of lipid-peroxidation chain reactions, while the incomplete rescue suggests that ferroptosis-related lipid peroxidation is an important but not exclusive component of WGN-induced redox injury.

A key issue addressed by this study is the position of c-Myc in WGN-associated redox-defense changes. c-Myc is generally recognized as an oncogenic transcription factor involved in proliferation, metabolism, mitochondrial activity, and oxidative stress adaptation [20,21,22]. Its relationship with ferroptosis is context dependent: c-Myc may increase oxidative and metabolic pressure, while it may also sustain antioxidant programs that protect tumor cells from ferroptosis-related injury [19]. In the present model, c-Myc overexpression partially restored GPX4 protein expression and alleviated WGN-induced viability loss, Fe^2+^ accumulation, and lipid ROS elevation, whereas c-Myc knockdown was accompanied by GPX4 reduction. These data support a functional association between c-Myc and GPX4-related antioxidant defense in A549 cells, but do not establish direct transcriptional regulation of GPX4 by c-Myc.

The SLC7A11 and ACSL4 responses provide additional molecular context for the ferroptosis-related phenotype. SLC7A11 downregulation may reduce the cystine/GSH supply required for GPX4 function, and ACSL4 upregulation may increase membrane lipid substrates vulnerable to peroxidation. These changes should be interpreted as supporting evidence rather than proof that a single c-Myc/GPX4 pathway fully accounts for WGN-induced injury. Future GPX4 rescue, ACSL4 knockdown, SLC7A11 functional experiments, GSH/GSSG quantification, and GPX4 activity measurements will be necessary to determine the relative contribution of each antioxidant and lipid-peroxidation component.

Although mitochondrial dysfunction and Caspase-related apoptosis were observed, these findings should be interpreted as cooperating redox-stress responses rather than an isolated apoptosis-only mechanism. WGN increased ROS, reduced mitochondrial membrane potential, and increased Caspase-related apoptotic markers, while NAC and Z-VAD-FMK each produced partial rescue. These results indicate that oxidative stress and apoptosis contribute to cytotoxicity, but they do not establish the temporal or causal relationship between mitochondrial injury and ferroptosis-related lipid peroxidation. The partial protection observed after inhibitor pretreatment is consistent with the involvement of more than one cell-injury pathway.

The in vivo and organoid findings strengthen the biological relevance of the observed response pattern but should be interpreted cautiously. In xenografts, WGN suppressed tumor growth and reduced c-Myc and *GPX4* expression, indicating that selected molecular changes observed in A549 cells were also detectable in vivo. Patient-derived organoids further reproduced decreased c-Myc/*GPX4* and increased ACSL4 in a three-dimensional model that better preserves tumor architecture and drug-response heterogeneity. However, neither the xenograft nor the organoid experiments included ferroptosis-specific functional rescue or tissue-level biochemical measurements of lipid peroxidation. Therefore, these models mainly support antitumor activity and selected redox-associated molecular trends rather than proving ferroptosis as the in vivo or organoid mechanism. Public transcriptomic cohorts should likewise be interpreted as supportive rather than causal evidence because they reveal associations among *MYC*, *GPX4*, ferroptosis-related states, proliferative programs, and clinical outcomes but cannot directly define transcriptional changes after WGN exposure or prove target engagement [28,29].

Several limitations should be considered when interpreting the mechanistic hierarchy and model scope. The present data support functional involvement of c-Myc in GPX4-associated ferroptosis sensitivity, but they do not prove that WGN directly targets c-Myc or that c-Myc directly binds the GPX4 promoter. The absence of ChIP-qPCR, dual-luciferase reporter assays, CETSA, SPR, GPX4 overexpression rescue, ACSL4 knockdown, SLC7A11 functional rescue, GSH/GSSG quantification, and GPX4 activity measurements limits the ability to define a direct regulatory chain or rank all ferroptosis-related components with certainty. The study also lacks a detailed time-course analysis of ROS accumulation, ferroptosis-related lipid peroxidation, and apoptosis, so the temporal order among these events remains unresolved. In addition, in vivo Fer-1 rescue and tissue-level measurements of MDA, 4-HNE, GSH/GSSG, labile iron, and antioxidant enzyme activity would be required to more directly evaluate ferroptosis in tumor tissues. Because the mechanistic experiments were mainly performed in A549 cells, which have specific genetic and redox characteristics including KEAP1/NRF2-related antioxidant adaptation, validation in additional NSCLC cell lines with distinct genetic backgrounds is required before extrapolating this c-Myc/GPX4-associated response to NSCLC more broadly [30].

Although the present study focused on c-Myc/GPX4-associated changes, other redox-defense pathways may also contribute to the WGN response. A549 cells exhibit KEAP1/NRF2-related antioxidant adaptation, and GPX4-independent systems, including the FSP1/AIFM2-CoQ10 pathway, can also suppress lipid peroxidation. Because these pathways were not directly measured, the observed response cannot be attributed exclusively to c-Myc/GPX4 signaling [13,30].

The weaker effect of WGN on BEAS-2B cells may be related to the lower basal oxidative pressure and different antioxidant-buffering dependence of non-cancer bronchial epithelial cells compared with A549 tumor cells. This interpretation remains speculative because the present study only used BEAS-2B as a normal-cell comparator and did not systematically analyze redox-buffering capacity in non-cancer cells.

At present, the antitumor development of WGN remains largely preclinical. Its clinical translation is limited by the need for additional pharmacokinetic, formulation, dose-optimization, safety, and efficacy data, as well as validation in additional NSCLC models. Therefore, the present findings should be viewed as preclinical evidence rather than direct clinical proof [23].

Overall, this study suggests that WGN suppresses A549-associated NSCLC phenotypes in association with ROS accumulation, ferroptosis-related lipid stress, and mitochondrial dysfunction-associated apoptosis. WGN treatment was associated with reduced c-Myc/GPX4 antioxidant protection, increased lipid ROS accumulation, and Fe^2+^-associated cellular injury. SLC7A11 downregulation and ACSL4 upregulation further support a ferroptosis-permissive molecular state. These findings provide a rationale for further evaluating c-Myc/GPX4 as a candidate biomarker axis of WGN responsiveness and for exploring combination strategies that reinforce lipid peroxidation in c-Myc-driven or GPX4-dependent NSCLC.

## 5. Conclusions

This study demonstrates that WGN suppresses A549-associated NSCLC malignant phenotypes in cell culture, nude mouse xenografts, and patient-derived NSCLC organoids. This effect is accompanied by ROS accumulation, decreased mitochondrial membrane potential, Caspase-related apoptosis, Fe^2+^ accumulation, increased lipid peroxidation, and reduced c-Myc/GPX4-associated antioxidant defense. WGN treatment decreased c-Myc and GPX4 protein levels, while SLC7A11 downregulation and ACSL4 upregulation provided additional ferroptosis-related molecular support. NAC, Z-VAD-FMK, and Fer-1 each produced partial rescue, and c-Myc overexpression and knockdown further supported a functional association between the c-Myc/GPX4-associated state and WGN-induced redox changes. Overall, WGN may suppress A549-associated NSCLC phenotypes in association with ROS accumulation, ferroptosis-related lipid injury, and mitochondrial dysfunction-associated apoptosis, although direct c-Myc-to-GPX4 regulation and broader NSCLC generalizability require further validation.

## Data Availability

The original contributions presented in this study are included in the article and Supplementary Materials. Public transcriptomic datasets analyzed in this study are available from the Gene Expression Omnibus under accession numbers GSE19188, GSE19804, GSE30219, and GSE31210. The raw data supporting the conclusions of this article, including uncropped Western blot images and numerical source data, are available from the corresponding author upon reasonable request.

## Supplementary Materials

The following supporting information can be downloaded at: https://www.mdpi.com/article/10.3390/antiox15070891/s1. Figure S1: MitoTracker analysis of mitochondrial membrane potential after WGN treatment; Table S1: RT-qPCR primer sequences; Table S2: c-Myc siRNA oligonucleotide sequences.

## Abbreviations

ACSL4	Acyl-CoA synthetase long-chain family member 4
ANOVA	Analysis of variance
BCA	Bicinchoninic acid
CCK-8	Cell counting kit-8
cDNA	Complementary DNA
CETSA	Cellular thermal shift assay
ChIP	Chromatin immunoprecipitation
CI	Confidence interval
DAPI	4′,6-Diamidino-2-phenylindole
DCFH-DA	2′,7′-Dichlorodihydrofluorescein diacetate
DMEM	Dulbecco’s Modified Eagle Medium
DMSO	Dimethyl sulfoxide
E-cadherin	Epithelial cadherin
EDTA	Ethylenediaminetetraacetic acid
FBS	Fetal bovine serum
FDR	False discovery rate
Fer-1	Ferrostatin-1
GAPDH	Glyceraldehyde-3-phosphate dehydrogenase
GPX4	Glutathione peroxidase 4
GSE	Gene Expression Omnibus series
GSH	Reduced glutathione
GSSG	Oxidized glutathione
H&E	Hematoxylin and eosin
4-HNE	4-Hydroxynonenal
HR	Hazard ratio
IHC	Immunohistochemistry
JC-1	Tetraethylbenzimidazolylcarbocyanine iodide
MDA	Malondialdehyde
MMP9	Matrix metalloproteinase 9
MTT	3-(4,5-Dimethylthiazol-2-yl)-2,5-diphenyltetrazolium bromide
MYC	Myelocytomatosis oncogene (c-Myc gene)
NAC	N-Acetylcysteine
NSCLC	Non-small cell lung cancer
PBS	Phosphate-buffered saline
PCNA	Proliferating cell nuclear antigen
PI	Propidium iodide
PVDF	Polyvinylidene difluoride
qPCR	Quantitative polymerase chain reaction
RhoNox-6	Rhodamine-based Fe^2+^ fluorescent probe
ROS	Reactive oxygen species
RPMI	Roswell Park Memorial Institute medium
RT	Reverse transcription
SD	Standard deviation
SDS-PAGE	Sodium dodecyl sulfate–polyacrylamide gel electrophoresis
SE	Standard error
siRNA	Small interfering RNA
SLC7A11	Solute carrier family 7 member 11
SPR	Surface plasmon resonance
TFRC	Transferrin receptor
WGN	Wogonin
Z-VAD-FMK	Benzyloxycarbonyl-Val-Ala-Asp(OMe)-fluoromethylketone
