# Supplementary material for: Wogonin Suppresses Non-Small Cell Lung Cancer Growth in Association with Oxidative Stress, c-Myc/GPX4 Downregulation and Ferroptosis-Related Responses
Source: Antioxidants (Basel). 2026 Jul 19;15(7):891. doi: 10.3390/antiox15070891 (PMC13403425; doi:10.3390/antiox15070891)
Supplement: Supplementary file 1 [file antioxidants-15-00891-s001.zip › antioxidants-4405971-supplementary.pdf]

Supporting Information

Wogonin Suppresses Non-Small Cell Lung Cancer Growth in Association with Oxidative Stress, c-Myc/GPX4 Downregula-tion and Ferroptosis-Related Responses

Figure S1. Related to the mitochondrial dysfunction analysis associated with Fig. 2F and G.

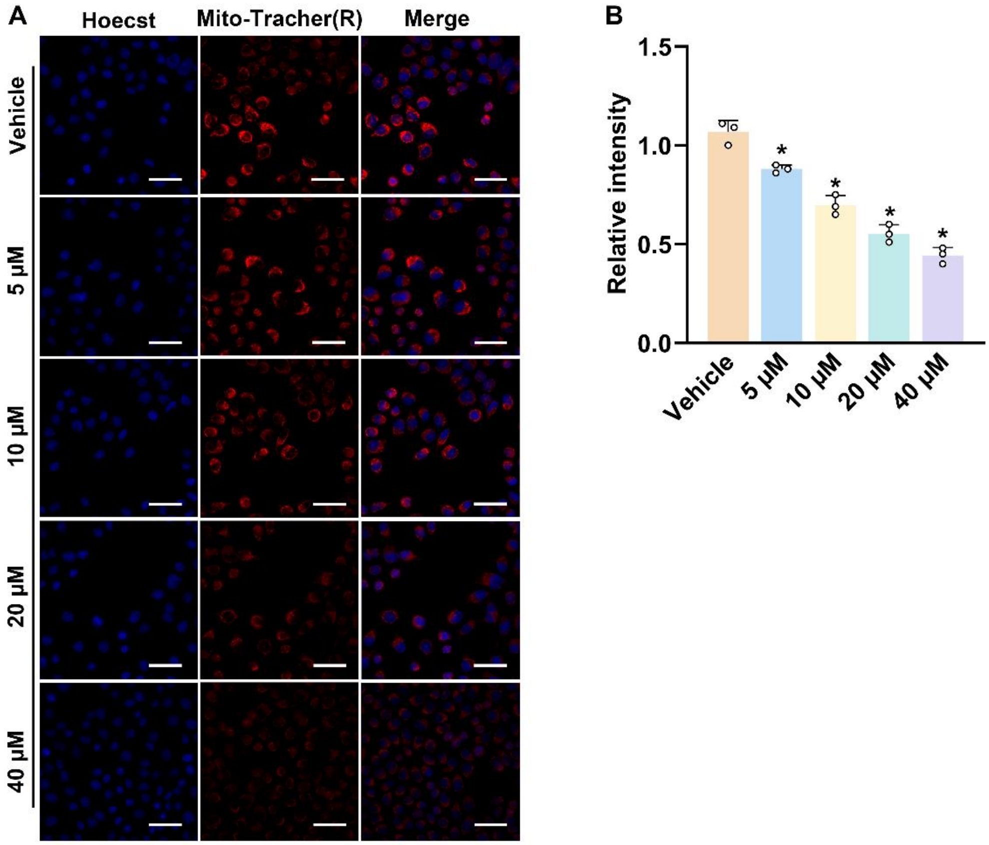

Fig. S1. MitoTracker Red staining further supports mitochondrial dysfunction in A549 cells after WGN exposure. A549 cells were treated with WGN (0, 5, 10, 20, and 40 µM) for 24 h. (A) Representative fluorescence images. (B) Quantification of relative fluorescence intensity. Data are presented as mean ± SD (n = 3). One-way ANOVA followed by Tukey's post hoc test; *P < 0.05 vs. Vehicle. Scale bar: 100 µm.

Figure S2. Related to the in vivo toxicity assessment associated with Fig. 7D.

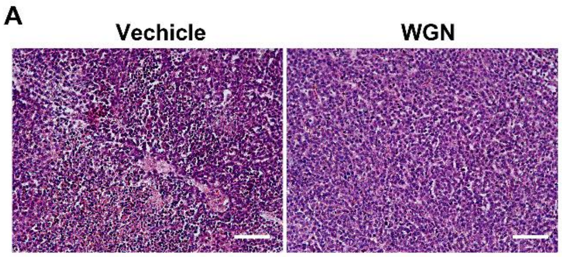

Fig. S2. H&E staining of xenograft tumor tissues used together with body-weight monitoring to assess in vivo toxicity under the current dosing regimen. Tumor sections from Vehicle- and WGN-treated mice (60 mg/kg/day, intraperitoneally for 8 days) showed no obvious abnormalities in tissue morphology. Scale bar: 50 μm (n = 6).

Supplementary Tables

SI Table 1. RT-qPCR primer sequences used in this study.

Gene	Forward primer（5′→3′）	Reverse primer（5′→3′）
c-MYC	GGCTCCTGGCAAAAGGTCA	CTGCGTAGTTGTGCTGATGT
SLC7A11	GGGCATGTCTCTGACCATCT	TCCCAATTCAGCATAAGACAAA
GPX4	ACAAGAACGGCTGCGTGGTGAA	GCCACACACTTGTGGAGCTAGA
ACSL4	CATCCCTGGAGCAGATACTCT	TCACTTAGGATTTCCCTGGTCC
GAPDH	AGAAGGCTGGGGCTCATTTG	AGGGGCCATCCACAGTCTTC
CASP3	GGAAGCGAATCAATGGACTCTGG	GCATCGACATCTGTACCAGACC

SI Table 2. c-Myc siRNA oligonucleotide sequences used in this study.

siRNA Name	Sense strand（5′→3′）	Antisense strand（5′→3′）	Description
MYC-Homo-1708	CUACGGAACUCUUGUGCGUTT	ACGCACAAGAGUUCCGUAGTT	Used for knockdown efficiency screening
MYC-Homo-1421	ACAACCGAAAAUGCACCAGTT	CUGGUGCAUUUUCGGUUGUTT	Used for knockdown efficiency screening
MYC-Homo-621	GCCCUCCUACGUUGCGGUCTT	GACCGCAACGUAGGAGGGCTT	Exhibited relatively high knockdown efficiency and was selected for subsequent experiments
MYC-Homo-853	CUGGCCUCCUACCAGGCUGTT	CAGCCUGGUAGGAGGCCAGTT	Used for knockdown efficiency screening
